# The protective effects of PI3K/Akt pathway on human nucleus pulposus mesenchymal stem cells against hypoxia and nutrition deficiency

**DOI:** 10.1186/s13018-020-1551-9

**Published:** 2020-01-28

**Authors:** DaSheng Tian, Jianjun Liu, Lei Chen, Bin Zhu, Juehua Jing

**Affiliations:** Department of Orthopaedics, The Second Affiliated Hospital of Anhui Medical University, 678#Fu Rong Road, Hefei, Anhui 230601 People’s Republic of China

**Keywords:** PI3K/Akt, Human nucleus pulposus-derived mesenchymal stem cells, Hypoxia and nutrition deficiency, Intervertebral disc degeneration

## Abstract

**Background:**

To study the effects of hypoxia and nutrition deficiency mimicking degenerated intervertebral disc on the biological behavior of human nucleus-derived pulposus mesenchymal stem cells (hNP-MSCs) and the role of PI3K/Akt pathway in the process in vitro.

**Methods:**

hP-MSCs were isolated from lumbar disc and were further identified by their immunophenotypes and multilineage differentiation. Then, cells were divided into the control group, hypoxia and nutrition deficiency group, the LY294002 group, and insulin-like growth factor 1 (IGF-1) group. Then cell apoptosis, the cell viability, the caspase 3 activity, and the expression of PI3K, Akt, and functional genes (aggrecan, collagen I, and collagen II) were evaluated.

**Result:**

Our work showed that isolated cells met the criteria of International Society for cellular Therapy. Therefore, cells obtained from degenerated nucleus pulposus were definitely hNP-MSCs. Our results showed that hypoxia and nutrition deficiency could significantly increase cell apoptosis, the caspase 3 activity, and inhibit cell viability. Gene expression results demonstrated that hypoxia and nutrition deficiency could increase the relative expression of PI3K and Akt gene and inhibit the expression of functional genes. However, when the PI3K/Akt pathway was inhibited by LY294002, the cell apoptosis and caspase 3 activity significantly increased while the cell viability was obviously inhibited. Quantitative real-time PCR results showed that the expression of functional genes was more significantly inhibited. Our study further verified that the above-mentioned biological activities of hNP-MSCs could be significantly improved by IGF1.

**Conclusions:**

PI3K/Akt signal pathway may have protective effects on human nucleus pulposus-derived mesenchymal stem cells against hypoxia and nutrition deficiency.

## Background

Intervertebral disc (IVD) degeneration appears to be the foremost cause of the back pain [1], which causes a tremendous economic burden to society [2]. Nucleus pulposus (NP) cells presenting in the intervertebral disc maintain the extracellular matrix [3]. The decrease in NP cell viability lead to the disturbing disc homeostasis, characterized by loss of extracellular matrix. Exogenous mesenchymal stem cells (MSCs) have shown an ability to regenerate disc cells and maintain the normal structure of degenerated IVD [4]. Recently, endogenous MSCs stimulation and recruitment is indicated to be an essential way to repair IVD degeneration [5]. Previous studies have confirmed that human degenerated and normal IVD contained human nucleus pulposus-derived mesenchymal stem cells (hNP-MSCs) [6–9] and hNP-MSCs fulfilled morphological, immunophenotypic, and differentiation definition criteria described by the International Society of Cell Therapy for mesenchymal stromal cells. These cells could retard the process of IVD degeneration [10]. The aim of endogenous hNP-MSCs therapy is to make hNP-MSCs differentiate into NP-like cells and promote disc cells maintaining metabolic balance and biomechanical functions of IVD. However, it is hard to maintain the number of the active hNP-MSCs under the adverse microenvironment in the degenerated IVD and leads to repair failure of endogenous hNP-MSCs [11].

The IVD obtains all essential nutrients through the cartilage endplate [12]. Some studies found that there were steep gradients in the concentration of nutrients across the IVD with oxygen level ranging from 1 to 5% [13]. With the processing of aging and degeneration, the supply of nutrients such as oxygen, glucose, and serum reduces significantly and even disappears, making the microenvironment more hypoxic and nutrition deficiency, which leads to metabolic disturbance and decrease of nucleus pulposus cells and nucleus pulposus progenitor cells [14]. Previous studies have investigated the single effect of hypoxia [15] and nutrition deprivation [16] presenting in the IVD on the biological activities of IVD cells, but microenvironment in the degenerated IVD is complex, consisting of hypoxia and nutrition deficiency. There have been no study that investigates whether hypoxia and nutrition deficiency are able to change the activities of the hNP-MSCs. Thus, in our present study, we investigated the effect of hypoxia (1% O_2_) and nutrition deficiency (no glucose and no fetal serum), mimicking the microenvironment in the degenerated IVD on the hNP-MSCs as in the previous study [17]. We hypothesized that microenvironment of hypoxia and nutrition deficiency may inhibit the biological activities of hNP-MSCs.

The phosphatidylinositol 3-kinase (PI3K)/Akt pathway plays a critical role in cell proliferation, apoptosis, and differentiation during physiological and pathological conditions [18, 19]. PI3K is a unique family of intracellular lipid kinases and Akt is a serine/threonine kinase. Once activated by different agents, PI3Ks change phosphatidylinositol 4,5-biphosphate (PIP2) into phosphatidylinositol 3,4,5-triphosphate (PIP3, [20]) and further activate Akt. Activated Akt modulates biological processes, including cell proliferation and apoptosis through interaction with downstream proteins. More importantly, PI3K/Akt signaling activation has shown protective effect on IVD degeneration. Activation of PI3K/Akt signaling increases SOX9 expression and activity, which induces expression of aggrecan in NP cells [21]. Insulin-like growth factor-1(IGF-1) promotes IVD cells proliferation by activating this pathway [22]. Many adverse factors contribute to IVD degeneration, such as adverse microenvironment, cytokines. Previous studies indicated that activation of PI3K/AKT signaling attenuated NP cells apoptosis induced by high-magnitude compression [23] or hyperosmotic conditions [24]. In addition, PI3K/AKT could protect NP cells against apoptosis induced by some cytokines, such as IL-1β [25] and TNF-ɑ [26]. Thus, we supposed that whether PI3K/Akt also played a protective effect on hNP-MSCs under hypoxia and nutrition deficiency.

In our present study, we speculated that hypoxia and nutrition deficiency could inhibit biological behavior of hNP-MSCs and PI3K/Akt pathway could protect hNP-MSCs from hypoxia and nutrition deficiency. We isolated cells from human degenerated lumbar disc and identified whether this type of cell fulfilled the definition criteria of MSCs established by an expert panel from the International Society for cellular Therapy (ISCT). Afterwards, we investigated the effects of hypoxia and nutrition deficiency on the biological behavior of hNP-MSCs in vitro, and studied the role of PI3K/Akt signaling in the process.

## Materials and methods

### Cell isolation and culture

All procedures performed in studies involving human participants were in accordance with the ethical standards of the institutional and/or national research committee and with the 1964 Helsinki declaration and its later amendments or comparable ethical standards. This research has been approved by the IRB of the authors’ affiliated institutions. Informed consent was obtained from all individual participants included in the study.

Our present study collected three disc samples from patients who underwent spine surgery for degenerative spine diseases. All data about the source of the disc material were showed in Table 1. Briefly, gel-like NP tissues taken from human degenerated IVD were carefully separated from the AF using a microscope under aseptic conditions. Thereafter, NP tissue was minced into small pieces before enzymatic digestion with 0.02 mg/ml collagenase type II (Gibco, USA) at 37 °C overnight. The suspension was centrifuged at 1500 rpm for 5 min and the supernatant was discarded. The remaining tissue and cells was cultured in standard MSC culture medium, consisting of Dulbecco’s modified Eagle’s medium-low glucose (HyClone, USA), 10% fetal calf serum (Gibco, USA), and 1% penicillin/streptomycin (Gibco, USA) at 37 °C in 5% CO_2_ for 24 h. Suspended cells and tissue were abandoned, and adherent cells were cultured in standard MSC expansion medium with complete replacement of the medium every 3 days. When adherent cells reached 80% to 90% confluence, they were subcultured at 1:3. Cells at passage 2 were used for subsequent experiments.

**Table 1:** Patients characteristics

	Age (year)	Gender	Modified Pfirrmann grading	Level
Case1	31	Female	III	L4–5
Case2	44	Female	VI	L5–S1
Case3	66	Male	IV	L5–S1

### Immunophenotype of hNP-MSCs

Cells were rinsed and suspended in PBS (Sigma, USA) at 1 × 10^5^ cells/ml. Further, 100 μl cell suspension solution was aliquoted per tube and was respectively incubated with a solution of CD34, CD73, CD45, CD90, CD105, and HLA-DR (eBioscience, USA) at room temperature for 30 min. Isotype control was used in each case. Cells were rinsed in PBS and suspended in 500 μl of PBS prior flow cytometry analyses.

### Multilineage differentiation

Mesenchymal Stem Cell Differentiation Medium (Cyagen Biosciences, Guangzhou, China) were purchased for differentiation assays according to the manufacturer’s instructions, which includes osteogenic differentiation, adipogenic differentiation, and chondrogenic differentiation. The cells were planted at 2 × 10^4^ cells/cm^2^ for osteogenic differentiation, 2 × 10^4^ cells/cm^2^ for adipogenic differentiation, and 5 × 10^5^ cells/ml for chondrogenic differentiation, respectively. The cells were then respectively stained with Alizarin Red, Oil Red O, and Alcian Blue. The entire stained areas were visualized under inverted microscopy.

### Preparation of four experimental groups

Cells, isolated from all three disc samples, were cultured and passaged. For each experiment, cells from three disc samples were selected for each assay. These cells were divided into four groups, including the control group (group1), the hypoxia and nutrition deficiency group (group2), the LY294002 group (group3), and IGF-1 group (group4). The cells in the control group were cultured under normoxia with standard MSC expansion medium (21% O_2_, 10% fetal calf serum, 5 mM glucose). The cells in the hypoxia and nutrition deficiency group were cultured with hypoxia and nutrition deficiency (1% O_2_, no fetal calf serum, no glucose). LY294002, a specific PI3K inhibitor, with a concentration of 30–50 μM, was shown to specifically inhibit PI3K activity without inhibiting other lipid and protein kinases [27]. The cells in the LY294002 group were added into 30 μmol/L LY294002 for 1 h before cultured under hypoxia and nutrition deficiency conditions. The cells in the IGF1 group were added into 200 μg/L IGF1, a PI3K activator, for 1 h before cultured under hypoxia and nutrition deficiency conditions.

### Apoptosis assay

hNP-MSCs were planted at 1 × 10^5^ cells/ml in 6-well plates, cultured under normal condition for 24 h, and then divided into above four groups and cultured for 24 h. Then harvested cells were incubated with 5 μl Annexin V-FITC and 5 μl PI (KeyGEN BioTECH, China) for 5 min at 37 °C in the dark and analyzed by Flow cytometry (BD, USA).

### CCK-8 assay

The viability of hNP-MSCs cultured in different conditions was evaluated by Cell-Counting Kit-8 (CCK-8). hNP-MSCs were planted at 5 × 10^4^ cells/ml in 96-well plates. These cells were cultured under normal condition for 1 day and then divided into above four groups and cultured for 1 day. Finally, the cells were incubated with 10 μl CCK-8 reagent (DOJINDO, Japan) for 4 h at 37 °C in the dark. Isotype group had no cells. The absorbance of different groups was measured at 450 nm using a Spectra MAX microplate reader.

### Caspase3 activity

hNP-MSCs were planted at 1 × 10^5^cells/ml in 6-well plates, cultured under normal condition for 24 h, and then divided into above four groups and cultured for 24 h. The caspase tetrapeptide substrate Ac-DEVD-pNA (Beyotime, China) was used to detect caspase 3 activity. Cells were washed with PBS and lysed with 100 μl of lysis buffer (Beyotime, China) on ice for 15 min. Then 10 μl of lysate was added to mixture solutions, consisting of 80 μl of reaction buffer and 10 μl of Ac-DEVD-pNA and incubated for 1 h at 37 °C. A microplate spectrophotometer was used to measure the optical density of free pNA.

### Gene expression of PI3K, Akt, aggrecan, collagen I, collagen II, stem cell-related genes (Oct4, Nanog, Jagged, and Notch1)

hNP-MSCs were planted at 1 × 10^5^ cells/ml in 6-well plates, cultured under normal condition for 24 h, and then divided into above four groups and cultured for 24 h. Total RNA was extracted from harvested cells using TRIReagent (Ambion, USA) according to the product instructions. Then, total RNA was reverse transcribed into the cDNA using a reverse-transcribed reagent (Takara, Japan). GAPDH was used as the reference gene. The obtained cDNA used in quantitative real-time PCR was analyzed using A SYBR Premix Ex Taq PCR kit (Takara, Japan) and LightCycler (Roche, Switzerland). Primers sequences of all genes were synthesized according to the software Premier 5.0, as shown in Table 1.

## Statistical analysis

All data were presented as means ±standard deviation (SD) of independent experiments (*n* = 3). All measurements were performed in triplicate. The SPSS version 17.0 software (IBM, USA) were used to analyze data. ANOVA with a post-hoc test were performed to analyze the results. *P* values < 0.05 were considered statistically significant (Table 2).

**Table 2:** Primers used in qRT-PCR

Genes	Sense primer	Antisense primer
Oct4	GTGAGAGGCAACCTGGAGAA	GAACCACACTCGGACCACAT
jagged	CGAGGACTATGAGGGCAAGA	CTTCAGGTGTGTCGTTGGAA
Notch1	GCCAGAGTGGACAGGTCAGT	ACACACACGCAGTTGTAGCC
Nanog	AGGCAAACAACCCACTTCTG	TCTGCTGGAGGCTGAGGTAT
Collagen I	CCTGGAAAGAATGGAGATGATG	ATCCAAACCACTGAAACCTCTG
Collagen II	GGTAAGTGGGGCAAGACTGTTA	TGTTGTTTCTGGGTTCAGGTTT
Aggrecan	GTCAGATACCCCATCCACACTC	CATAAAAGACCTCACCCTCCAT
PI3K	ACCAGCACTGCCTCCTAAAC	TCTTCATCATCTTCCACCAGTG
Akt	ACTCTTTCCAGACCCACGACC	CAAAGAAGCGATGCTGCATG

## Results

### Isolation and characterization of hNP-MSCs

**Fig. 1:**
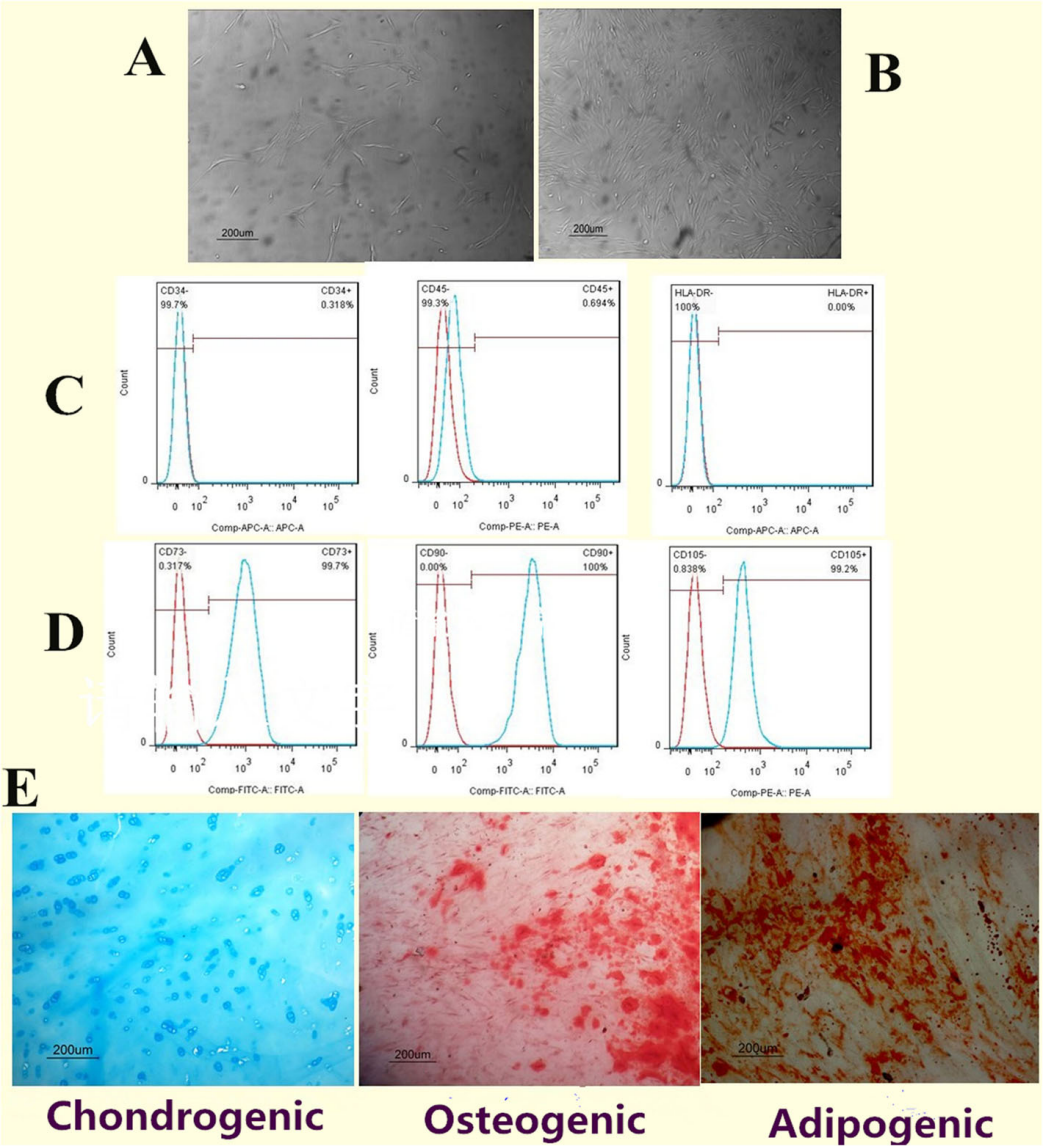
Primary hNP-MSCs present short spindle-shape (**a**). P2 hNP-MSCs presented the characteristic spindle-shape and grew in spiral formation (**b**). These cells were highly positive for CD73, CD90, CD105, and negative for CD34, CD45, HLA-DR (**c, d**). hNP-MSCs possessed osteogenic, adipogenic, and chondrogenic differentiation (**e**)

Primary cells were observed after 3–5 days of initial cell culture and presented short spindle-shape (Fig. 1a). The cells grew significantly faster when cultures were passaged and grew in spiral formation and also consistently presented the characteristic spindle-shape (Fig. 1b). These cells isolated from degenerated IVD were highly positive for CD73, CD90, and CD105, and were negative for CD34, CD45, and HLA-DR (Fig. 1c, d). Alizarin Red staining showed that cells formed mineralized nodules. Oil Red O staining revealed that cells produced intracellular lipid vacuoles. Alcian Blue staining indicated that cells exhibited sulfated proteoglycan (Fig. 1e). These results suggested that these cells fulfilled the definition criteria of MSCs [28] and hNP-MSCs were successfully obtained from human degenerated NP.

### Hypoxia and nutrition deficiency increased the gene expression of PI3K and Akt

**Fig. 2:**
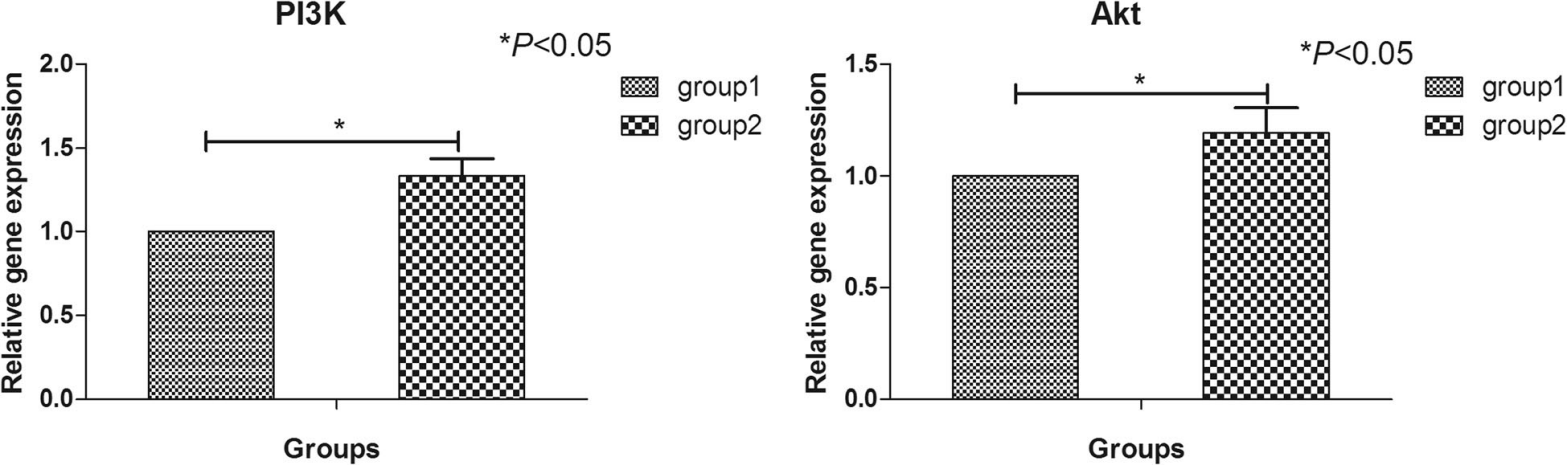
The relative gene expression of PI3K and Akt in normal condition, hypoxia and nutrition deficiency condition evaluated by qRT-PCR. *p < 0.05 indicated significant difference between groups

The relative gene expression of PI3K and Akt in group 2 was notably higher than that in group 1 (*P* < 0.05), which indicated that PI3K/Akt pathway could be involved in the process under hypoxia and nutrition deficiency (Fig. 2).

### Hypoxia and nutrition deficiency inhibited the proliferation of hNP-MSCs and inhibiting PI3K by LY294002 could aggravate this inhibiting effect while activating of PI3K by IGF-1 could improve the biological activity

**Fig. 3:**
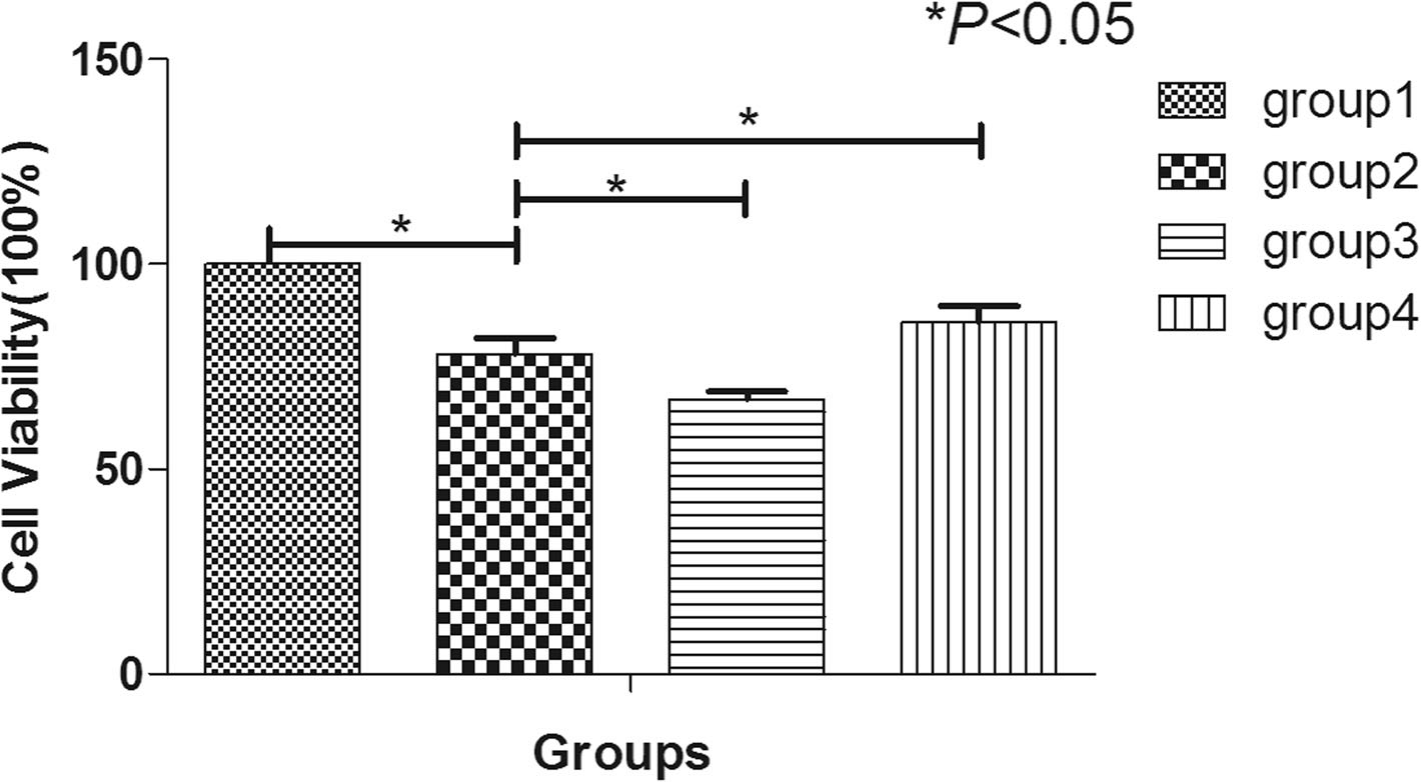
The viability of hNP-MSCs cultured in normal condition (group 1), hypoxia and nutrition deficiency condition (group 2), the LY294002 condition (group 3), and the IGF-1 condition (group 4) evaluated by cell-counting kit-8 (CCK-8). *p < 0.05 indicated significant difference between groups

The cell proliferation of group 2 was notably lower than that of group 1 (*P* < 0.05), which indicated that hypoxia and nutrition deficiency could inhibit the proliferation of hNP-MSCs. Meanwhile, after blocking PI3K by LY294002, the proliferation in group 3 was significantly lower than that in group 2 (*P* < 0.05); however, after activating of PI3K by IGF1, the proliferation in group 4 was obviously higher than that group 2 (*P* < 0.05) (Fig. 3).

### Hypoxia and nutrition deficiency induced apoptosis of hNP-MSCs and inhibiting PI3K by LY294002 could aggravate this effect while activating of PI3K by IGF-1 could attenuate the apoptosis

**Fig. 4:**
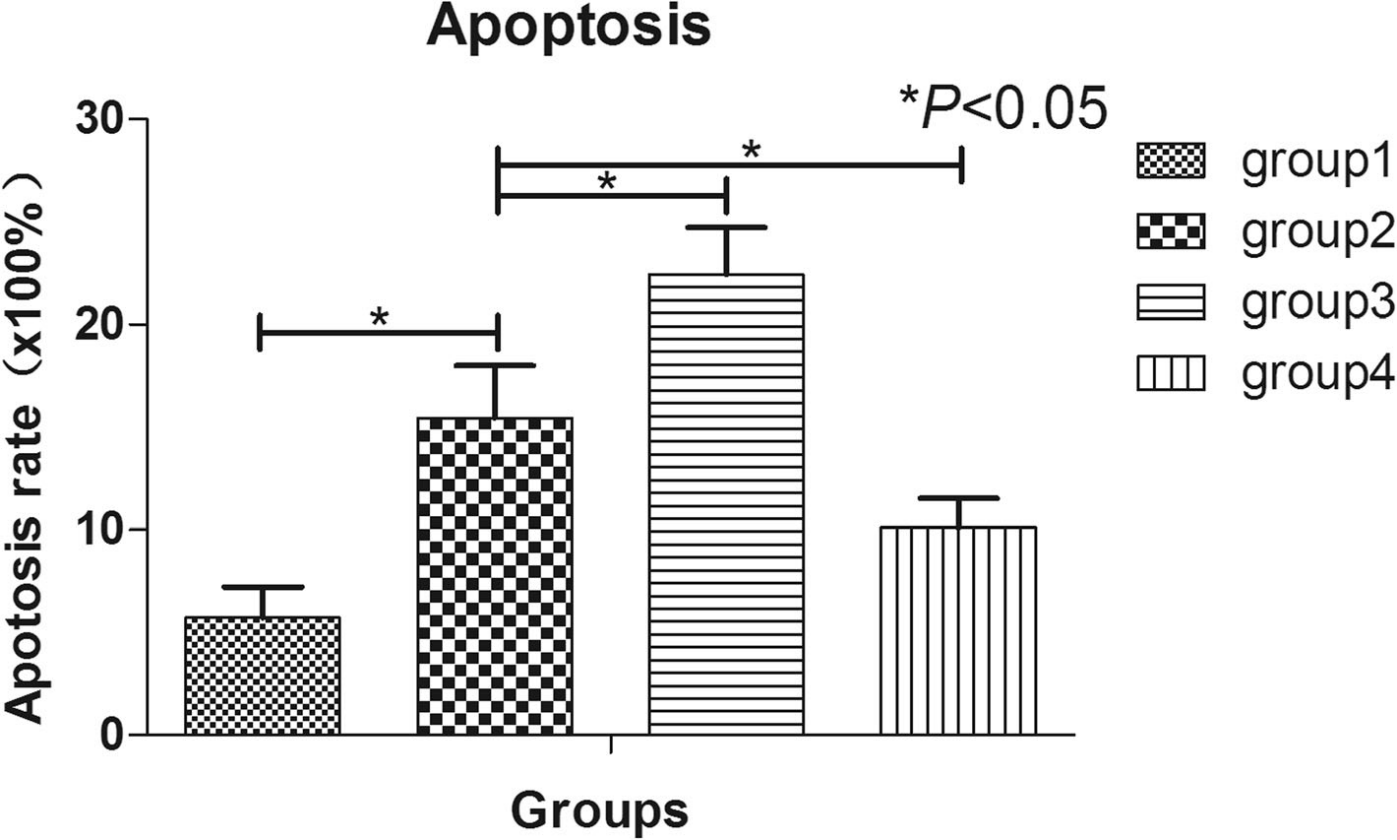
The apoptosis of hNP-MSCs cultured in normal condition (group 1), hypoxia and nutrition deficiency condition (group 2), the LY294002 condition (group 3), and the IGF-1 condition (group 4) evaluated by flow cytometry. *p < 0.05 indicated significant difference between groups

The cell apoptosis of group 2 was significantly higher than that of group 1 (*P* < 0.05), which indicated that hypoxia and nutrition deficiency could induce the apoptosis of hNP-MSCs. Meanwhile, after blocking PI3K by LY294002, the apoptosis of group 3 was significantly higher than that of group 2 (*P* < 0.05); however, after activating of PI3K by IGF1, the apoptosis of group 4 was obviously lower than that of group 2 (*P* < 0.05) (Fig. 4).

### Hypoxia and nutrition deficiency increased caspase 3 activity and inhibiting PI3K by LY294002 could aggravate this effect while activating of PI3K by IGF-1could inhibit the caspase3 activity

**Fig. 5:**
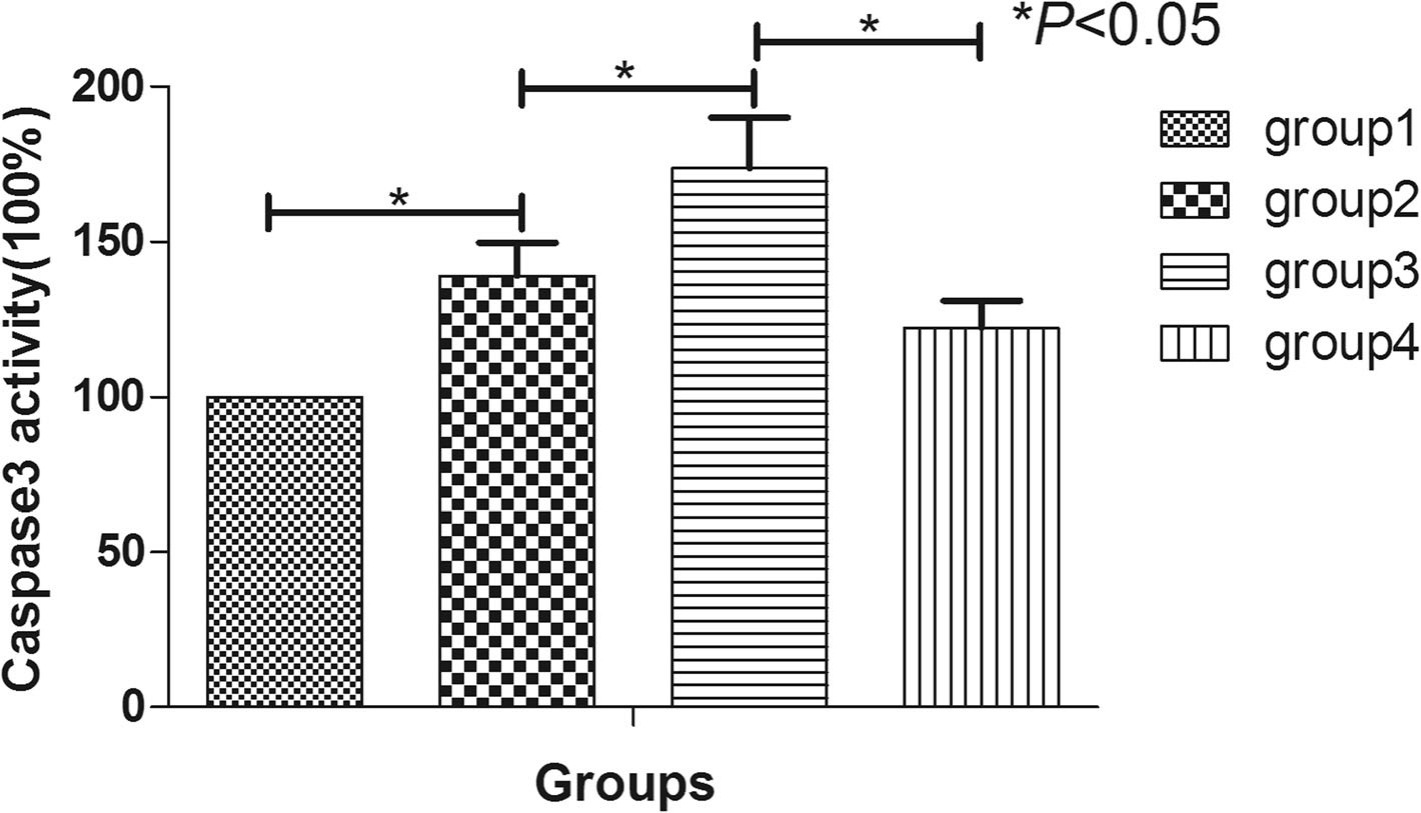
The caspase 3 activity of hNP-MSCs cultured in normal condition (group 1), hypoxia and nutrition deficiency condition (group 2), the LY294002 condition (group 3), and the IGF-1 condition (group 4) evaluated by Ac-DEVD-pNA. *p < 0.05 indicated significant difference between groups

The optical density value of group 2 was significantly higher than that of the group 1 (*P* < 0.05), which indicated that hypoxia and nutrition deficiency could increase caspase3 activity. Meanwhile, after blocking PI3K by LY294002, the value of group 3 was significantly higher than that of group 2 (*P* < 0.05); however, after activating of PI3K by IGF1, the value of group 4 was obviously lower than that of group 2 (*P* < 0.05) (Fig. 5).

### Hypoxia and nutrition deficiency downregulated the expression of functional genes and stem cell-related genes and blocking PI3K by LY294002 could aggravate this effect while activating of PI3K by IGF-1 could upregulate the expression levels

**Fig. 6:**
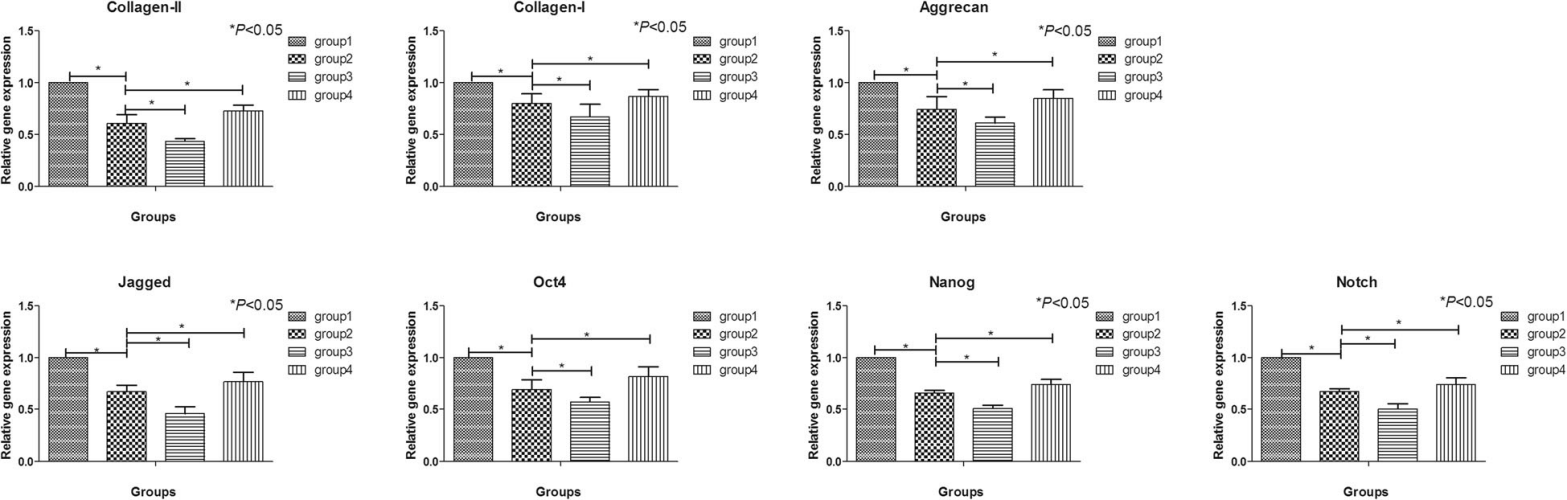
The relative gene expression of PI3K, Akt, aggrecan, collagen I, and collagen II in hNP-MSCs cultured in normal condition (group 1), hypoxia and nutrition deficiency condition (group 2), LY294002 condition (group 3), and IGF-1 condition (group 4) evaluated by qRT-PCR. *p < 0.05 indicated significant difference between groups

The relative expression of functional genes and stem cell-related genes in group 2 was significantly lower than that of group 1 (*P* < 0.05), which indicated that hypoxia and nutrition deficiency could inhibit the gene expression of functional genes and stem cell-related genes in hNP-MSCs. Meanwhile, after blocking PI3K by LY294002, the relative expression level of group 3 was significantly lower than that of group 2 (*P* < 0.05); however, after activating of PI3K by IGF1, the relative expression level of group 4 was obviously higher than that of group 2 (*P* < 0.05) (Fig. 6).

## Discussion

The nucleus pulposus is the largest avascular tissue in the human body and nutrients diffuse across the cartilage endplate to reach disc cells and disc cells thus consume glucose and oxygen to generate energy to maintain cellular metabolism [29]. The microenvironment becomes hypoxic and nutrition deficient with the processing of degeneration. Hypoxia and nutrition deficiency have been respectively shown to inhibit viability and proliferation of nucleus pulposus cells and NP stem cells. But whether hypoxia and nutrition deficiency synergy have similar effects on hNP-MSCs has, until now, remained unknown. This is the first study to investigate whether hypoxia and nutrition deficiency, mimicking the complex conditions in the degenerated disc, could result in detrimental effects on hNP-MSCs.

In our present study, we cultured the hNP-MSCs in the hypoxia and nutrition deficiency condition in vitro, which mimicked the disc degenerative microenvironment. Trial results showed that the hypoxia and nutrition deficiency inhibited proliferation of hNP-MSCs; what is more, the detrimental microenvironment increased the apoptosis rate and enhanced the caspase 3 activity. Our data suggested that hypoxia and nutrition deficiency could induce apoptosis and inhibit the proliferation of hNP-MSCs. Aggrecan, collagen II, and collagen I were used as representative of the IVD matrix. PCR results showed that hNP-MSCs cultured in hypoxia and nutrition deficiency had decreased expression of aggrecan, collagen type II and I, indicating that hypoxia and nutrition deficiency could lead to metabolic disturbance in hNP-MSCs. hNP-MSCs expressed stem cell-related genes (Oct4, Nanog, Jagged, and Notch1) [30] and hypoxia and nutrition deficiency downregulated the expression levels of these genes. Previous study found that hypoxia inhibited the viability and proliferation of NP-MSCs, but promoted the chondrogenic differentiation of NPMSCs [15]. These results were a little different with our study. The microenvironment in the IVD is complex and presents hypoxia, nutrition deficiency, and so on. Thus, biological behavior of cells in the hypoxia and nutrition deficiency conditions may differ with that in the hypoxia conditions. Hypoxia and nutrition deficiency could not only inhibit proliferation and matrix synthesis but also promote the apoptosis of hNP-MSCs, which may explain why these hNP-MSCs could not maintain the stability of IVD.

Dysregulation of various signaling pathways has been shown to be involved in the disc degeneration [31]. PI3K/Akt signaling plays a critical role in cell proliferation, apoptosis, and differentiation during physiological and pathological conditions [18, 19]. PI3K/Akt pathway has been shown to protect nerve cells from cerebral infarction [32] and protect cardiomyocytes from hypoxia-induced cell injury [33]. More importantly, targeting this pathway has been shown to execute protective role in IVDD [34]. Our study found that hypoxia and nutrition deficiency could upregulate the expression of PI3K/Akt, which indicated that PI3K/Akt pathway could be involved in the process under hypoxia and nutrition deficiency condition.

LY294002 is regarded as a specific inhibitor of PI3K [27] and have been shown to reverse the protective of PI3K/Akt pathway [35]. Thus, in the study, we further assessed the effect of blocking PI3K/Akt pathway by LY294002 on the biological activities of hNP-MSCs. Our study observed that treatment of hNP-MSCs with LY294002 under hypoxia and nutrition deficiency aggravated the decrease in cell proliferation and matrix gene expression. Blocking PI3K/Akt pathway by LY294002 could also increase cell apoptosis. Additionally, treatment of hNP-MSCs with LY294002 aggravated the decrease in the expression of stem cell-related genes.

Insulin-like growth factor 1(IGF-1) has a significant protective effects against IVD degeneration [36]. Some studies found that IGF-1 dramatically increased collagen and aggrecan content in NP cells by activating the PI3K/Akt pathway [37]. Meanwhile, IGF-1 can promote cell proliferation in human IVD cells through activation of this pathway [22]. Thus, in our study, we further assessed the effect of activation of PI3K/Akt pathway by IGF-1 on the biological activities of hNP-MSCs. Our study observed that addition of IGF-1 to hNP-MSCs under hypoxia and nutrition deficiency could meliorate the decrease in cell proliferation and matrix gene expression, and also inhibit cell apoptosis. Additionally, treatment of hNP-MSCs with IGF-1 improves the decrease in the expression of stem cell-related genes.

All above results showed that inhibition of PI3K/Akt pathway could aggravate the harmful effect induced by hypoxia and nutrition deficiency condition while activation of PI3K/Akt pathway could meliorate the inhibiting effect, which demonstrated that PI3K/Akt signal pathway has protective effects on hNP-MSCs against hypoxia and nutrition deficiency. However, the downstream signaling pathway of PI3K/Akt still remains unclear. mTOR, a key protein of downstream signal pathway of PI3K/Akt [38], acts as major signal of cell growth and is involved in proliferation, protein translation, and the anti-apoptosis process [24, 39]. Previous studies suggested that PI3K/Akt/mTOR signaling pathways was involved in protection against IL-1β [25] or serum deprivation-induced IVD cells apoptosis [40] and activation of mTOR suppressed activated caspase3. In our present study, PI3K/Akt was involved in the hypoxia and nutrition deficiency conditions and the results showed that activation of PI3K/Akt decreased caspase 3 activity, so we could make an assumption that PI3K/Akt/mTOR/caspase 3 signaling pathway may be involved in this process and needs further work to demonstrate it.

There are some limitations in our study. Firstly, the effect of hypoxia and nutrition deficiency on hNP-MSCs was performed in vitro and in 2D culture medium, so further studies need to be carried out in vivo and 3D culture system. Secondly, our study mainly investigated the relative expression of genes to indirectly reflect the changes appeared in hypoxia and nutrition deficiency microenvironment, lacking of protein evidence to directly reflect the changes. Finally, the precise mechanism of PI3K/Akt pathway in regulating hNP-MSCs in response to hypoxia and nutrition deficiency remains unknown.

## Conclusions

PI3K/Akt signal pathway may have protective effects on human nucleus pulposus mesenchymal stem cells against hypoxia and nutrition deficiency. We could protect endogenous stem cells from nutrient deficiency-induced death by activating the pathway and provide seeds to repair IVD.

## Data Availability

The datasets used and/or analyzed during the current study are available from the corresponding author on reasonable request.

## Abbreviations

hNP-MSCs: Human nucleus pulposus mesenchymal stem cells
IGF-1: Insulin-like growth factor-1
ISCT: International Society for cellular Therapy
IVD: Intervertebral disc
MSCs: Mesenchymal stem cells
NP: Nucleus pulposus
